# Supplementary material for: Inhibiting stromal Class I HDACs curbs pancreatic cancer progression
Source: Nat Commun. 2023 Dec 6;14:7791. doi: 10.1038/s41467-023-42178-6 (PMC10700526; doi:10.1038/s41467-023-42178-6)
Supplement: Supplementary file 3 — Description of Additional Supplementary Files [file 41467_2023_42178_MOESM3_ESM.pdf]

Description of Additional Supplementary Files

Supplementary Data 1: Top 25 TF candidates that potentially regulate activation-induced and Ent-repressed genes in PSCs, and the expression of these TFs in PSC samples.

Supplementary Data 2: Lists of genes transcriptionally regulated in PSC activation and functionally related to myofibroblast identity, proliferation and lipid metabolism.

Supplementary Data 3: Top 25 enriched genes in fibroblast subpopulations identified by scRNA-seq.

Supplementary Data 4: Sequences of primers used in RT-qPCR.
