# Supplementary material for: Inhibiting stromal Class I HDACs curbs pancreatic cancer progression
Source: Nat Commun. 2023 Dec 6;14:7791. doi: 10.1038/s41467-023-42178-6 (PMC10700526; doi:10.1038/s41467-023-42178-6)
Supplement: Supplementary file 8 — Reporting Summary [file 41467_2023_42178_MOESM8_ESM.pdf]

Corresponding author(s): Ronald M Evans

Last updated by author(s): Oct 9, 2023

Reporting Summary

Nature Portfolio wishes to improve the reproducibility of the work that we publish. This form provides structure for consistency and transparency in reporting. For further information on Nature Portfolio policies, see our Editorial Policies and the Editorial Policy Checklist.

Statistics

For all statistical analyses, confirm that the following items are present in the figure legend, table legend, main text, or Methods section.

n/a	Confirmed	
☐	☒	The exact sample size (*n*) for each experimental group/condition, given as a discrete number and unit of measurement
☐	☒	A statement on whether measurements were taken from distinct samples or whether the same sample was measured repeatedly
☐	☒	The statistical test(s) used AND whether they are one- or two-sided<br>*Only common tests should be described solely by name; describe more complex techniques in the Methods section.*
☒	☐	A description of all covariates tested
☒	☐	A description of any assumptions or corrections, such as tests of normality and adjustment for multiple comparisons
☐	☒	A full description of the statistical parameters including central tendency (e.g. means) or other basic estimates (e.g. regression coefficient) AND variation (e.g. standard deviation) or associated estimates of uncertainty (e.g. confidence intervals)
☐	☒	For null hypothesis testing, the test statistic (e.g. *F*, *t*, *r*) with confidence intervals, effect sizes, degrees of freedom and *P* value noted<br>*Give P values as exact values whenever suitable.*
☒	☐	For Bayesian analysis, information on the choice of priors and Markov chain Monte Carlo settings
☒	☐	For hierarchical and complex designs, identification of the appropriate level for tests and full reporting of outcomes
☒	☐	Estimates of effect sizes (e.g. Cohen's *d*, Pearson's *r*), indicating how they were calculated

Our web collection on statistics for biologists contains articles on many of the points above.

Software and code

Policy information about availability of computer code

Data collection	qPCR was performed using a CFX384 detection system (Bio-Rad). Immunofluorescent images were acquired using a LSM 710 laser scanning confocal microscope system (Zeiss) or a VS120-L100 virtual slide system (Olympus). High-throughput sequencing experiments in RNA-seq, ATAC-seq and scRNA-seq were run on HiSeq 2500, NextSeq 500 and NovaSeq systems (Illumina), respectively. Immunoassay was carried out with a Bio-Plex 200 System (Bio-Rad). Western blot images were acquired using a ChemiDoc XRS+ system (Bio-rad). Ultrasound imaging was performed with a Vevo 3100 system (Fujifilm VisualSonics). Histology images were acquired with a VS120-L100 Virtual Slide System (Olympus). Mass spectrometry data was collected with a TSQ Quantiva mass spectrometer (Thermo). Cell sorting and flow cytometry were respectively carried out on a FACSAria Fusion sorter (BD) and a FACSCantoII or LSRII analyzer (BD). For in vitro proliferation/survival assay, GFP and Titer-Glo luminescent signals were measured by an Envision (Perkin Elmer) plate reader.

Data analysis	All data analyses were performed using common softwares. qPCR data was analyzed with CFX Maestro 2.3 software (Bio-rad, v5.3.022.1030). For RNA-seq analysis, raw sequencing data was processed by CASAVA (Illumina, v1.8.2) and aligned to mouse or human reference genome using the STAR aligner (v2.5.1b); differential gene expression analysis was performed using Cuffdiff (v2.2.1); hierarchical clustering was carried out using Cluster software (v3.0) and visualized by Java Treeview (v3.0). GSEA analysis was performed with GSEA software (v4.0.3). GO term and TF binding site enrichment analysis was performed with online database DAVID (v6.8) and Enrichr, respectively. For ATAC-seq analysis, raw reads were mapped to mouse reference genome with Bowtie2 (v2.3.4.3); peak calling and motif analysis were performed with HOMER software (v4.0). For scRNA-seq analysis, raw reads were aligned to mouse reference genome using Cell Ranger (10x Genomics, v4.0); marker identification and UMAP generation were conducted with the R package Seurat (v3). Western blotting images were quantified by Image Lab (Bio-rad, v5.2.1). Mass spectrometry data was analyzed with Skyline software (v21.1). Images in histology analysis on tissue sections were quantified with Image J (v1.54f). Flow cytometry data were analyzed with FlowJo software (v10.7.1). Statistical analysis was performed using Prism software (v 10.0.2). Patient data were retrieved from the database of The Cancer Genome Atlas (TCGA-PAAD) via Survexpress software (v2.0) or extracted from the NCBI Gene Expression Omnibus dataset GSE71729.

For manuscripts utilizing custom algorithms or software that are central to the research but not yet described in published literature, software must be made available to editors and reviewers. We strongly encourage code deposition in a community repository (e.g. GitHub). See the Nature Portfolio guidelines for submitting code & software for further information.

Data

Policy information about availability of data

All manuscripts must include a data availability statement. This statement should provide the following information, where applicable:

- Accession codes, unique identifiers, or web links for publicly available datasets
- A description of any restrictions on data availability
- For clinical datasets or third party data, please ensure that the statement adheres to our policy

RNA-seq, ATAC-seq and scRNA-seq data generated in this study are deposited in the Sequence Read Archive (SRA) database of NCBI with accession number PRJNA524175 (https://www.ncbi.nlm.nih.gov/bioproject/PRJNA524175). The patient dataset of stromal gene expression (GSE71729) can be accessed from the GEO database of NCBI (https://www.ncbi.nlm.nih.gov/geo/query/acc.cgi?acc=GSE71729). The TCGA-PAAD dataset can be accessed from GDC data portal (https://portal.gdc.cancer.gov/projects/TCGA-PAAD). Source data are provided with this paper.

Research involving human participants, their data, or biological material

Policy information about studies with human participants or human data. See also policy information about sex, gender (identity/presentation), and sexual orientation and race, ethnicity and racism.

Reporting on sex and gender	None.
Reporting on race, ethnicity, or other socially relevant groupings	None.
Population characteristics	None.
Recruitment	None.
Ethics oversight	None.

Note that full information on the approval of the study protocol must also be provided in the manuscript.

Field-specific reporting

Please select the one below that is the best fit for your research. If you are not sure, read the appropriate sections before making your selection.

☒ Life sciences ☐ Behavioural & social sciences ☐ Ecological, evolutionary & environmental sciences

For a reference copy of the document with all sections, see nature.com/documents/nr-reporting-summary-flat.pdf

Life sciences study design

All studies must disclose on these points even when the disclosure is negative.

Sample size	Given the effect magnitudes in the study was unknown, sample sizes were not determined by power analysis but estimated based on experience and existing literature on specific animal models (Shi et al Nature, 569, 131-135).
Data exclusions	All samples and animals designated for the study were included.
Replication	Experiments in this study were mostly conducted with at least three independent or biologically independent replicates, except for ATAC-seq and a limited set of RNA-seq experiments, in which independent duplicates were used. Animal studies were performed with 4-18 animals per arm, depending on animal models and assays. All data from the experiments was included in the analysis, with total or representative data presented in the paper. All attempts at replication were successful.

Randomization	Animals were randomly enrolled for transplantation experiments or therapeutic treatments with no significant difference in body weight or in health (by visual assessment) between study arms.
Blinding	Experiments in the study were not blinded because the same researcher conducted the experiments and collected and analyzed the data, except for histopathological grading and ultrasound measurement of orthotopic transplants.

Reporting for specific materials, systems and methods

We require information from authors about some types of materials, experimental systems and methods used in many studies. Here, indicate whether each material, system or method listed is relevant to your study. If you are not sure if a list item applies to your research, read the appropriate section before selecting a response.

Materials & experimental systems

n/a	Involved in the study
☐	☒ Antibodies
☐	☒ Eukaryotic cell lines
☒	☐ Palaeontology and archaeology
☐	☒ Animals and other organisms
☒	☐ Clinical data
☒	☐ Dual use research of concern
☐	☒ Plants

Methods

n/a	Involved in the study
☒	☐ ChIP-seq
☐	☒ Flow cytometry
☒	☐ MRI-based neuroimaging

Antibodies

Antibodies used

Antibodies against α-SMA (Santa Cruz, sc-32251, 1:100, Clone 1A4, Lot A1218) and Ki67 (Abcam, ab15580, 1:500, Lot GR3196372-1), and fluorochrome-conjugated secondary antibodies against mouse (Alexa Fluor Plus 555; Invitrogen, A32727, 1:2000, Lot WA316324) and rabbit IgG (Alexa Fluor 647; Invitrogen, A27040, 1:2000, Lot 222638) were used in immunofluorescent staining. Antibodies against pSTAT3 (Cell Signaling, 9145, 1:1000, Clone D3A4, Lot 34,), STAT3 (Cell Signaling, 12640, 1:1000, Clone D3Z2G, Lot 4), and α-tubulin (Sigma, T6199, 1:1000, Clone DM1A, Lot 029M4842V), and secondary antibodies against rabbit (Santa Cruz, sc-2004, 1:5000, Lot H0913) and mouse IgG (Santa Cruz, sc-2005, 1:5000, Lot E3113) were used in Western blotting. The anti-LIF antibody used for LIF neutralization (Clone D25) was provided by Tony Hunter (Salk). Primary antibodies against CK19 (Epitomic, AC-0073, 1:2000, Clone EP72, Lot E0051705), α-SMA (Santa Cruz, sc-32251,1:1000, Clone 1A4, Lot A1218), Ki67 (Abcam, ab15580, 1:5000, Lot GR3196372-1), Fabp4 (Abcam, ab92501, 1:5000, Clone EPR3579, Lot GR3375349-14) and CD8a (Invitrogen, 14-0195-82, 1:100, Clone 4SM16, Lot 2297451) were used in IHC staining, together with secondary antibody solutions provided in the staining kits (Cell Signaling, 8125 and 13079). Fluorochrome-conjugated anti-CD45 (BV510; Biolegend, 103138, 1:200, Clone 30-F11, Lot B296479), anti-Epcam (Alexa Fluor 647; Biolegend, 118212, 1:200, Clone G8.8, Lot B264359), and anti-Pdpn (APC-Cy7; Biolegend, 127418, 1:400, Clone 8.1.1, Lot B253983) antibodies were used in FACS.

Validation

All primary antibodies used in this study are commercially available and validated by the manufacturers, except for the anti-LIF antibody validated by Tony Hunter's group and reported in Shi et al Nature, 569, 131-135 (2019). The validation information for the commercial antibodies is as follows.

α-SMA (Santa Cruz, sc-32251): IF (immunofluorescence) and IHC (P) (immunohistochemistry, paraffin-embedded) are listed under "Applications", and mouse under "Species Reactivity/Detection" (https://www.scbt.com/p/alpha-actin-antibody-1a4).

Ki67 (Abcam, ab15580): ICC/IF (immunocytochemistry/immunofluorescence) and IHC-P (immunohistochemistry, formalin/PFA-fixed, paraffin-embedded) are listed under "Tested applications", and mouse under "Species reactivity" (https://www.abcam.com/products/primary-antibodies/ki67-antibody-ab15580.html).

pSTAT3 (Cell Signaling, 9145), STAT3 (Cell Signaling, 12640): Western blotting is listed under "Application" in "Product Usage Information", and mouse (M) and human (H) under "Species Reactivity" (https://www.cellsignal.com/products/primary-antibodies/phospho-stat3-tyr705-d3a7-xp-rabbit-mab/9145?_requestid=661012; https://www.cellsignal.com/products/primary-antibodies/stat3-d3z2g-rabbit-mab/12640).

α-tubulin (Sigma, T6199): Western blot analysis is stated and referenced under "Application", and mouse and human α-tubulin are stated to be recognized under "Specificity" (https://www.sigmaaldrich.com/US/en/product/sigma/t6199).

CK19 (Epitomic, AC-0073): Paraffin (standing for paraffin-embedded IHC) is listed under "Reactivity" (https://www.cellmarque.com/antibodies/EP/2588/Cytokeratin-19_EP72), and the application in mouse tissue is referenced in Shi et al Nature, 569, 131-135 (2019).

Fabp4 (Abcam, ab15580): IHC-P is listed under "Tested applications", and mouse under "Species reactivity" (https://www.abcam.com/products/primary-antibodies/fabp4-antibody-epr3579-ab92501.html).

CD8a (Invitrogen, 14-0195-82): IHC is listed under "Applications" along with 6 publications, and mouse is listed under "Species Reactivity" and under "Published species" (https://www.thermofisher.com/antibody/product/CD8a-Antibody-clone-4SM16-Monoclonal/14-0195-82).

CD45 (Biolegend, 103138), Epcam (Biolegend, 118212), Pdpn (Biolegend, 127418): FC (flow cytometry) is listed under "Application"

and noted with "Quality tested" (referring to that "each lot of this antibody is quality control tested"), and mouse is listed under "Verified Reactivity" (https://www.biolegend.com/en-us/products/brilliant-violet-510-anti-mouse-cd45-antibody-7995?GroupID=BLG1932; https://www.biolegend.com/en-us/products/alexa-fluor-647-anti-mouse-cd326-ep-cam-antibody-4973?GroupID=BLG5748; https://www.biolegend.com/en-us/products/apc-cyanine7-anti-mouse-podoplanin-antibody-13646?GroupID=BLG5772).

Eukaryotic cell lines

Policy information about cell lines and Sex and Gender in Research

Cell line source(s)	The mouse PDAC cell line FC1245 and organoid lines mT9, hm1A and hT3 were provided by David Tuveson (Cold Spring Harbor Laboratory) and Herve Tiraic (University of California, San Diego). The mouse PDAC cell line p53 2.1.1 was provided by Eric Collison (UCSF). The human PDAC cell lines MIA PaCa2 (CRL-1420), PSN1 (CRM-CRL-3211) and Panc1 (CRL-1469) were acquired from ATCC. The GFP-labelled FC1245 line and the mouse CAF lines (imCAF1, 2) were generated in this study. The human CAF cells ONO, YAM and hPSC1 were provided by Atsushi Masamune (Tohoku University).
Authentication	Cell lines from ATCC were authenticated by STR profiling by ATCC. Other cell lines were monitored for morphology and growth rate when in culture, and applied to experiments at limited passages (<10).
Mycoplasma contamination	Cell lines used in this study were tested negative for mycoplasma contamination in routine tests during cell culture.
Commonly misidentified lines (See ICLAC register)	None.

Animals and other research organisms

Policy information about studies involving animals; ARRIVE guidelines recommended for reporting animal research, and Sex and Gender in Research

Laboratory animals	Mice used in the study were imported from vendors and/or bred in-house. Mice were housed at a temperature (22± 1°C) and humidity (45–65%) controlled environment with a 12-h light-dark cycle. Wild-type C57BL/6J male mice (8-12 weeks, the Jackson Laboratory) were used as PSC donors and hosts of orthotopic transplantation with FC1245 cells. Wild-type FVB/NJ male mice (9-10 weeks, the Jackson Laboratory) were used as hosts of subcutaneous co-implantation of CAFs (imCAF1) and PDAC cells (p53 2.1.1). KPf/fC mice with Rosa26luc/luc in the FVB/NJ background were used as a GEMM (Shi et al., Nature, 569, 131-135), and both female and male were enrolled in therapeutic treatment around the age of 25 days. Athymic nude mice (female, 4-6 week) were used as hosts of subcutaneous implantation of PDX and sacrificed when tumor were beyond 2,000 mm3 (Ng et al., J Control Release, 352, 1134-1143). No mice in the study carried tumors exceeding 2,000 mm3, the tumor size limit approved by IACUC.
Wild animals	None.
Reporting on sex	Findings in the paper is not sex-dependent. Sex-disaggregated data of therapeutic treatments are included in Source Data.
Field-collected samples	None.
Ethics oversight	All procedures were conducted in compliance with relevant institutional and national guidelines. All animal protocols were approved by the Institute of Animal Care and Use Committee (IACUC) at the Salk Institute and the University of Arizona (where animal studies of the Translational Genomic Research Institute are performed).

Note that full information on the approval of the study protocol must also be provided in the manuscript.

Dual use research of concern

Policy information about dual use research of concern

Hazards

Could the accidental, deliberate or reckless misuse of agents or technologies generated in the work, or the application of information presented in the manuscript, pose a threat to:

No	Yes	
☒	☐	Public health
☒	☐	National security
☒	☐	Crops and/or livestock
☒	☐	Ecosystems
☒	☐	Any other significant area

Experiments of concern

Does the work involve any of these experiments of concern:

No	Yes	
☒	☐	Demonstrate how to render a vaccine ineffective
☒	☐	Confer resistance to therapeutically useful antibiotics or antiviral agents
☒	☐	Enhance the virulence of a pathogen or render a nonpathogen virulent
☒	☐	Increase transmissibility of a pathogen
☒	☐	Alter the host range of a pathogen
☒	☐	Enable evasion of diagnostic/detection modalities
☒	☐	Enable the weaponization of a biological agent or toxin
☒	☐	Any other potentially harmful combination of experiments and agents

Flow Cytometry

Plots

Confirm that:

☒ The axis labels state the marker and fluorochrome used (e.g. CD4-FITC).

☒ The axis scales are clearly visible. Include numbers along axes only for bottom left plot of group (a 'group' is an analysis of identical markers).

☒ All plots are contour plots with outliers or pseudocolor plots.

☒ A numerical value for number of cells or percentage (with statistics) is provided.

Methodology

Sample preparation

FACS isolation of stromal fibroblasts was performed with single cell suspensions prepared from tumors from KPf/fC mice. Tumors were minced with a razor blade and incubated with agitation for 1 h with 20 ml of freshly made digestion buffer (DMEM with 1 mg/ml collagenase IV, 1 mg/ml hyaluronidase, 0.1% soybean trypsin inhibitor, 50 U/ml DNase I and 0.125 mg/ml dispase). Dissociated tumors were filtered through 100 µm cell strainers, processed with ACK lysis buffer (Gibco), and resuspend as single cell susupensions. Staining was performed using a standard protocol with fluorochrome-conjugated antibodies against CD45 (BV510; Biolegend, 103138, 1:200), Epcam (Alexa Fluor 647; Biolegend, 118212, 1:200) and Pdpn (APC-Cy7; Biolegend, 127418, 1:400).

Apoptosis analysis was performed with cell line samples prepared as single cell suspensions, and stained with 7-AAD and Annexin V staining using APC-conjugated Annexin V detection kit (eBiosciences, 88-8007-72) and 7-AAD (eBiosciences, 00-6993-50), following the manufacturer's instructions.

Cell cycle distribution analysis was performed with cell line samples prepared as single cell suspensions,using Click-IT EdU Alexa Fluor 647 Flow Cytometry Assay Kit (Invitrogen, C10424) and DNA dye Hoechst 33342 (10 µg/ml), following the manufacturer's instructions.

Instrument

Cell sorting was conducted with a FACSAria Fusion sorter (BD). Flow cytometry analysis was performed with a FACSCantoII or LSR-II cytometry (BD).

Software

Data were collected with FACSDiva (BD) and analyzed with FlowJo (BD).

Cell population abundance

The enrichment of stromal fibroblasts (Pdpn+) were confirmed by qPCR analysis detecting the specific expression of fibroblast markers (e.g. Pdpn, Col1a1) and minimal amplifications of markers for other cell types (e.g. Epcam, Ptprc, Pecam) in the sorted fibroblast population.

Gating strategy

All gating schemes were performed with the initial step for gating single cells sequentially by FSC-A/SSC-A, FSC-A/SSC-W and FSC-A/FSC-W. For isolation of stromal fibroblasts, live single cells were gated based on DAPI negativity from the single cells; CD45-negative cells gated from the live single cells; tumor/epithelial cells marked by Epcam and stromal fibroblasts marked by Pdpn were gated from the CD45-negative cells. For EdU-labeling assay, cells in G1, S and G2/M phases were gated based on DAPI and EdU-APC signals from the single cells. For Annexin V/7-AAD assay, cells were gated in quadrants based on Annexin V-APC and 7-AAD signals from the single cells. All compensations if applicable were carried out using samples stained with single fluorophores. Gatings were based on fluorescent minus one (FMO) controls or fluorescent intensity. Samples were incubated with mouse Fc block prior to antibody staining in order to reduce nonspecific staining.

☒ Tick this box to confirm that a figure exemplifying the gating strategy is provided in the Supplementary Information.
